# Breaking Through Barriers and Building Disaster Mental Resilience: A Case Study in the Aftermath of the 2015 Nepal Earthquakes

**DOI:** 10.3390/ijerph16162964

**Published:** 2019-08-17

**Authors:** Alisha KC, Connie Cai Ru Gan, Febi Dwirahmadi

**Affiliations:** Centre for Environment and Population Health, School of Medicine, Griffith University, Nathan QLD 4111, Australia

**Keywords:** Nepal, earthquake, resilience, disaster, mental health, barriers, preparedness

## Abstract

Introduction: Nepal was hit by two devastating earthquakes in 2015 that disrupted its socio-economic system and shattered many lives, resulting in increased mental health issues during the post-earthquake phase. Disasters can have severe mental health impacts on the affected population, making it necessary to enhance resilience within communities and to help them to adapt well in the face of adversities. From these earthquakes, this study looks to identify measures needed to develop community mental resilience for disaster preparedness in Nepal. Method: We conducted this research using the qualitative case study method and thematic analysis (TA). Result: Several activities were carried out by organizations to support the psycho-social aspects of communities, but were challenged by existing barriers. After considering the present context, this study presents five recommendations for mental resilience and also suggests the utilization of existing resources, such as faith-based organizations and teachers in the communities. Despite the considerable impact, communities demonstrate their own resilience, to some extent, through the culture of sharing and helping each other. Conclusions: A firm commitment is required from the government to enhance resilience by mainstreaming mental health in all areas of disaster management and planning.

## 1. Introduction

Disasters continue to threaten harm to people, both physically and mentally, worldwide. A report by the Centre for Research on the Epidemiology of Disasters stated that a total of 315 disaster events happened in 2018, affecting 68.5 million people in 141 countries and incurring economic losses of US$131.7 billion [1].

Climate changes are also forecasted to increase the frequency and intensity of disasters in the future [2]. Apart from deaths, injuries, and socio-economic disruption, psychological distress and mental health impacts in the aftermath of disasters are not uncommon. Moreover, mental illness is highly stigmatized among people and many cultures and even has severe and life-changing consequences compared to any physical illness [3].

For our research focus, we examined two major devastating earthquakes of magnitude 7.8 and 7.6 that hit Nepal on 25 April and 12 May in 2015, respectively. This tragedy affected 8.5 million people [4], killed over 8900, displaced 450,000 [4], and left more than 17,000 injured [5]. The earthquakes severely damaged national assets, disrupted physical and social systems, and left a deep scar amongst the many who endured severe consequences.

According to the country’s National Disaster Report of 2017, more than 80% of Nepal’s population is at risk of being affected by natural disasters, such as floods, landslides, earthquakes, fire, storms, and hail. In terms of the likelihood of such events, Nepal falls amongst the 20 most disaster-prone countries in the world and ranks 11th in the world for earthquakes, as shown by high seismic activities in and around the country. Furthermore, Nepal ranks fourth with respect to vulnerability to climate change. In fact, the capital city Kathmandu is at the highest risk for a massive impact from a severe earthquake despite similar seismic activities in 21 cities around the world [6].

Nepal has one of the most impoverished mental health care systems of any country, with only a share of 0.08% of its total health budget, and has less than two psychiatrists per million people, and even fewer clinical psychologists [7]. Mental health services are only integrated as a small part of general health care. The leading role for Mental Health and Psychosocial Support (MHPSS) had been played by non-government organizations (NGOs) throughout history, but their efforts are unsupported by ruling governments, thus reducing the sustainability of programs [8]. A decade-long civil conflict, political instability, the absence of a proper focal body for mental health services, minimal budget allocation, low awareness on mental health, the lack of skilled human resources for mental health, and a near absence of community-level mental health services have collectively led to a service gap among many who require treatment [9,10,11].

Although various studies have recently focused on mental health impacts within the earthquake-affected population of Nepal [4,12,13], one study in 2010 showed the scarcity of related literature for Nepal on the promotion of psychosocial well-being, psychosocial support, and overall psychological and mental health [14]. After the 2015 earthquakes, several studies emerged in the field of mental health and disaster. In fact, one study evaluated MHPSS responses and their long-term implications [8], while another focused on the coordination of activities, the leadership of MHPSS programs, as well as service delivery [15]. Some studies have attempted to answer issues concerning service utilization and mental health outcome [16], social cures for post-traumatic stress [17], and religious interventions [18]. Another recent study explored the mental health impact from earthquakes and resilience amongst earthquake-affected villages, stating that 46% of survivors exhibited moderate to high resilience [19]. Even though post-traumatic impacts and psychological resilience has been an emerging field for a decade [20], societal resilience in terms of earthquakes is a much understudied area. This present study is the first to explore various aspects of mental resilience building grounded by the reality of the 2015 earthquakes in Nepal. It fills the gap in the literature by providing potential recommendations for policy development and necessary measures to reduce the impact of disasters on mental health.

### 1.1. Disasters and Mental Health Issues

In 2018 the number of deaths (11,804) caused by disasters globally fell versus the 2008–2017 yearly average of 67,572 deaths [1]. This drop in deaths reflects widespread improvement in disaster response capacities [21], yet the affected population is still increasing [22]. Disasters are more frequent than ever before and larger numbers of people are impacted, due to low resilience and high vulnerabilities [22]. Although a disaster has a pervasive and penetrating shock on the affected population [23], the impact on mental health is greater and lasts longer within a resource-constrained setting [24,25], as survivors undergo a delayed recovery process [26].

Various research suggests that people who are exposed to extreme traumatic events like disasters are at risk of suffering from psychological problems such as depression, anxiety, post-traumatic stress disorder (PTSD), and other unspecified types of stress [20,27]. The duration and severity of psychological distress is related to the prolonged suffering of mental health problems. Disruption of the social system, family and property losses, an inability to maintain a healthy lifestyle, and chaos all can have a tremendous effect on both physical and mental health [28]. The meta-analysis of Dai et al. includes 46 articles that show the incidence rate of PTSD among earthquake survivors is 23.7%, which is nearly 1 in 4 [29]. However, the majority of disaster-related impacts begins with stress and, with adequate support provided on time, the effect could be diluted and progress back to a healthy adaptation, rather than any damaging illness [30].

### 1.2. The Mental Resilience Approach for Disaster Preparedness

People who are exposed to one or several traumatic life-threatening events in their lifespan may have mental health impacts. Stress is a part of everyday life and becoming resilient does not mean non-exposure to extreme stress, as it comes along with the challenges of daily life [31,32]. The inevitable, intense, chronic, and overwhelming types of stress may exacerbate or give rise to anxiety, depression, and PTSD. Reducing stressors and providing resources to get back into the normal swing of things can dissolve the short-term impact and allow for a better level of functioning [33]. Evidence has shown a close association between disaster resilience building and mental health preparedness. It is argued that resilience is the critical factor for recovery from such psychological trauma related to a natural disaster as it increases the ability to cope with a stressful situation, to revive a healthy life, and to manage stress adequately [18].

The word resilience is attained from the Latin word “resilire”, which means to jump back or leap back [34], and is similarly used across various literature in the notion of “bouncing back” [35]. Holling (1973) is mentioned as the first to use the concept of resilience in ecological literature [34,36]. In the socio-ecological definition, resilience is defined as a system’s ability to take in disturbances and retain the same level of function when enduring changes [36]. Community resilience from the psychosocial aspect is described as community adaptation when there is maximum wellness, mental and behavioural health, functioning, and quality of life in population constituents [37].

The American Psychological Association defines resilience as a process of adapting well when presented with adverse circumstances or significant stressors [31]. Viewpoints on resilience mainly comprise the ability to react positively to stress or disturbances [2]. Resilience is not a stable outcome that is reached and remains constant; in fact, it is an ongoing development process when facing adversities and adapting to them [38]. The result of resilience is wellness, which relates to a high level of mental and behavioural health, functioning, and quality of life; however, a myriad of underlying problems could impact the quality of life [33]. The measurement of resilience is still a highly debated issue and usually revolves around two things, as follows: Recovery, which is the ability to come out of difficulties, and sustainability, which means being able to walk past adversities [2]. Many studies have focused on psychological or behavioural and mental health impacts seen within various affected communities aside from physical impact [39,40], but not many have focused on mental resilience to natural disasters. On looking closely into different studies, the two terms of mental resilience and psychological resilience are used interchangeably when discussing the mental health impact. Likewise, terminologies including community resilience building, coping strategies, adaptive capacities, and disaster mental health preparedness all aim to make communities mentally prepared for any future disaster and to deal with the impact through resistance, adaptation, or by overcoming the impact.

## 2. Research Methodology

The aim of this study is to identify the gaps in building community mental resilience and to develop a recommendation for preparedness in order to achieve better mental health outcomes.

### 2.1. Study Design

This study uses a qualitative case study research method to allow for an in-depth understanding of the phenomenon of interest in its natural settings [40] and from relevant viewpoints [41]. We took the 2015 earthquakes in Nepal as our case study, because various efforts and activities were drawn from both government and non-government levels in response to the impacts, thus giving an overview of the current situation and practical efforts in Nepal.

#### 2.1.1. Data Collection Method

The literature review method was carried out to form a basis for study, followed by secondary data collection, and key informants’ interviews. Data collection started with a global literature search on state-of-art mental resilience and the current situation in Nepal, followed by key informants’ interviews over their 2015 earthquakes’ experiences.

##### Literature Review

We mainly searched the ProQuest Psychology Database, PsycINFO, and the MEDLINE (PubMed) database, along with various other databases. The key search words were the following: (mental resilience OR psychological resilience) AND (disaster OR earthquake) AND Nepal; (mental resilience OR psycho* resilience) AND (Strateg* OR Approach*) AND Disaster; (Community resilience) AND (psychological OR mental health) AND (disaster impact OR disaster consequence*). Search results were only limited to the English language. References from significant articles were also screened and reviewed. Grey literature was obtained from Google scholar, the United Nations for Disaster Risk Reduction (UNDRR) website, reports from various international organizations, and from key organizations. Most of the reports from different local organizations of Nepal were obtained from different websites through Google search and networking.

##### In-Depth Semi-Structured Interviews

We conducted semi-structured in-depth interviews with members of organizations working in the area of mental-health during the post-earthquakes phase, which were based in Nepal. Initially, various web searches were made to identify organizations working in the area of mental health in Nepal. We obtained reports and documents from the 2015 earthquakes that included information on mental health related activities carried out by different organizations. From the initial search results, the identified organizations were contacted via e-mail addressed provided in their respective websites. The organizations that were approached were of both local and foreign origin, with a functional office based in Nepal. It was required that organizations were involved in supporting mental health aspects of earthquake-affected communities. Organizations ranged from a mixture of government, non-government, international non-government organizations, and nonprofit-humanitarian organizations. A total of 15 potential organizations were approached via e-mail explaining the nature and scope of the study, of which 12 responded by confirming their possible participation and nominating a suitable candidate from their organization. Thus, twelve possible interview participants were provided information through a participant information sheet sent along with a consent form via e-mail; however, only ten were included. The following inclusion criteria, including (i) actively involved in mental health activities carried out by their organization during post-earthquakes phase (ii) have access to internet, (iii) based in Nepal, and (iv) ability to speak English or Nepali language, were used in the study. Participants excluded were those that expressed their limitation to provide valuable input because of the minimal role of their organization towards mental health impacts; hence, their involvement in our area of interest. The interview guide was prepared based on the research aim and involved questions related to activities done by them during the 2015 earthquakes in the area of mental health and the contextual barriers identified by them. After the clarity on the nature of the study and the participant’s role was achieved, consent was taken and the interview date and time was finalized. Participants were contacted by Skype and interviews were recorded using the Skype audio recorder. The interview date and time were rescheduled when needed and participant comfort was ensured throughout the interview.

Interview Questions

1. What was the impact of the 2015 earthquakes on the mental or psychological health of affected communities?
2. What did your organization do to assist communities in coping up with psychological impact in the aftermath of the earthquakes? How was it carried out? Who were involved?
3. Regarding steps taken to assist affected communities, what went well, what went wrong, and what were the main lessons learned?
4. What were the contributing factors and barriers identified while assisting communities to cope with the impact on mental/psychological health?
5. What do you think should be done to make communities well prepared and adaptive for any future disaster?

#### 2.1.2. Data Analysis

We conducted a verbatim transcription to capture all relevant data from the interview. We then analyzed the data using thematic analysis (TA), which is the commonly used method for data analysis in a qualitative study [40], by identifying, analyzing, and reporting themes within the data [42]. The data analysis process involves generating raw data, familiarizing data, writing and re-writing ideas, generating code and identifying data, finding themes and mapping, and, lastly, interpreting the data [43]. Concept mapping is used to organize and interpret ideas during data analysis. Research rigor is maintained by methodological triangulation [40] and conflicting ideas are resolved through discussions.

#### 2.1.3. Ethics

Ethical clearance was obtained from the Griffith University Human Research Ethics Committee at first (reference number: 740/2018), followed by ethical clearance (reference number: 527/2018) from the Nepal Health Research Council (NHRC). Written informed consent was obtained, after clarity over the purpose and nature of the study was ensured amongst participants before the interview, through the Participants Information Sheet (PIS).

### 2.2. Case Study: The 2015 Nepal Earthquakes

#### 2.2.1. General Impacts and Disaster Response Operations

**Figure 1:**
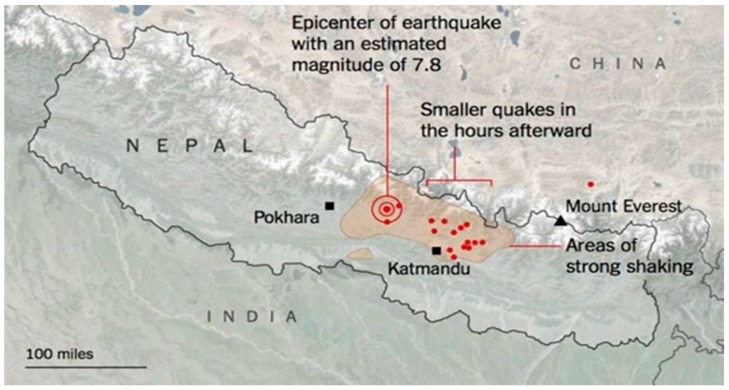
2015 Earthquake epicenter [44].

The first major earthquake was on 25 April at 11:56 (local time), registering a magnitude of 7.8, with its epicenter at Barpak VDC, which is 80 km away from the capital city Kathmandu. Up until the end of 2015, 421 aftershocks of magnitude 4 or greater occurred and four aftershocks of magnitude over 6.0 were noted. The second big earthquake happened 17 days later, on 12 May, with its epicenter at Sindupalchowk district; its magnitude was 7.6. This earthquake impacted 31 out of 75 districts in Nepal, of which 14 endured damages severe enough to be listed as crisis areas that demanded maximum assistance. The map of Nepal as Figure 1 showed below marks the epicenter of the first major earthquake and areas that endured maximum damages.

The earthquakes resulted in significant impacts on almost all sectors, including social (houses, health, education, human settlements, and cultural heritages), industry, agriculture, infrastructure, tourism, and many more; all of which shook the national economy. An additional 2.5–3.5% of Nepal’s population were pushed into poverty [45]. The Post-Disaster Needs Assessment (PDNA) estimated that NPR 669 billion (approximately US$6.8 billion) would be required to recover from the damage across all sectors.

Rural and remote communities were already living under deprivation before the earthquakes, which reflected the limitation of the livelihood system’s flexibility to sustain damages from significant catastrophes [46]. Furthermore, during the earthquakes, their only resources for recovery were destroyed [46] and, due to the remoteness of their location, services were not provided for several weeks afterwards [47]. The destruction of the health infrastructure and the already limited human resources led to greater difficulties in the provision of essential services [9]. The country was also still recovering from the impact of a decade-long Maoist insurgency and thus the 2015 earthquakes followed by an economic blockade and political conflicts worsened the complication.

#### 2.2.2. Mental Health Implications of the 2015 Nepal Earthquakes

Four months after the earthquakes, a survey [4] was conducted through representative sampling among survivors, who had higher estimates of depression (34%), anxiety (34%), suicidal thoughts (11%), and harmful alcohol drinking (20%). The rate for depression and anxiety was higher than WHO estimates (15–20%) within 12 months following humanitarian emergencies [4]. Another survey, conducted on 500 direct survivors in Bhaktapur, one of the worst-hit areas in Nepal, showed that close to 50% exhibited symptoms of psychiatric illness [13]. Commonly identified disorders included depression, stress, anxiety, and somatoform disorders. A recent study stated that 24.10% of adult survivors had PTSD 10 months after the earthquakes [48]. The three worst-hit districts of Kathmandu, Gorkha, and Sindupalchowk showed evidence that one in every three adults experienced symptoms of depression and distressful anger, one out of five indulged themselves in harmful drinking, and one out of ten had suicidal thoughts [4]. A study mentioned that the Nepal Police Force reported a 41% rise in suicidal thoughts versus three months before the event and revealed a 10.9% prevalence of suicidal thoughts in two of the worst-hit areas, Sindupalchowk and Gorkha, four months after the earthquakes [12].

## 3. Results

### 3.1. Mental Health Impacts After the Earthquakes

In the study, interview participants shared the mental impact of the earthquake from their own experience and perspective. The concerns of mental impacts were raised mainly on primary survivors who endured maximum unexplainable losses and emotional challenges. “*Those who lost their family and house and who could not gather resources to cope … had to worry on how to survive on top of everything that happened …. there has been a toxic effect*, *maybe for a lifetime*”. —Organization 7. People possessing higher values recover earlier as opposed to those with low resources who are unable to proceed with repairs, reconstruction, or relocation and who may take years to recover [49].

As mentioned by the participants, the earthquakes impacted the mental health of all age and socio-economic groups in different ways. However, the vulnerable groups identified were mainly children, elderly, and those from low socio-economic groups. The impact was mentioned to be greater on those who lost close family members and houses and who were under extreme deprivation of resources for their livelihood. In children, extreme fear and panic during the frequent aftershocks was reported. “*Children would run out of tents and panic even from the slightest noise*; *we struggled a lot to normalize them as they would get out of control in no time*”. —Organization 5

Participants reported some form of emotional challenges faced by workers without any severe consequences. In general, several issues such as emotional disturbances, stress, anxiety, sleep disturbances, depression, and anger were commonly reported within the affected communities. “*Within one year usually 400–500 mental illnesses would be seen …. but after the earthquakes within 9 VDCs (Village Development Committee) we diagnosed approximately 2500 mental illness cases*”.

Suicidal thoughts or harmful coping behaviour with alcohol and tobacco consumption were commonly identified patterns by participants when visiting earthquake-affected communities. Relapse of illness cases is a commonly noticed issue after an earthquake, as mentioned by a study in 2010 [50]. Participants also expressed concerns regarding the rising number of relapse cases in the post-earthquakes phase. Although a variety of mental health issues were identified amongst the affected Nepalese communities, PTSD (which is highly linked to disaster [27,51]) was less reported by participants in this study compared to anxiety and depression. Some studies noted that the prevalence of PTSD was 28.5% [25] and 18.5% [52] amongst affected groups. This disparity could be because of the difficulty in diagnosing PTSD [53] or the overestimation of mental illness [14]. Furthermore, PTSD prevalence is suggested to change with time [52]. Although mental impacts from the earthquakes are apparent, more rigorous studies to get the correct estimates of PTSD and other issues are needed.

### 3.2. An Overview of Mental Health-Related Actions and Resources After the Earthquakes

Various activities carried out by organizations were directed to both individuals as well as at the community levels. Psychological first aid (PFA) and psychosocial support (PSS) were the two frequently mentioned terms across all interviews and were mentioned as the primary services under the World Health Organization’s MHPSS guidelines. As outlined in the interviews, various services carried out by organizations ranged from communication and empathy sharing to counselling, referral, psychological therapies, and trauma healing sessions based on the needs of affected groups. Local practices were fostered in various ways to bring lives back to normal, such as keeping children in a friendly space, engaging in drawing, playing, involving adults and the elderly in religious or devotional songs, conducting cultural programs, or engaging in exercise. Psychosocial support was made available to the communities in many ways, either in the form of meeting basic needs, including physical needs, or with communication or reassurances through established hotline services and radio programs.

Participants mentioned that human-resource strengthening and capacity-building were significantly carried out by the organizations through awareness programs and training. They reported the acting organizations were under-resourced to serve the widely scattered and dispersed communities, because the extent of the impact was unimagined, despite that a good level of disaster awareness was present. Based on the practical experience during 2015 earthquakes, participants realized communities had minimal information about earthquakes or their consequences and the authorities lacked practical experiences. Despite these shortcomings, we identified the organizations that were able to respond immediately within 3–4 days by maximizing their efforts to support fourteen severely affected districts (combining the areas covered by all organizations).

This study identified that the next step taken by organizations was training staff and community members in psychological first aid and psychosocial support, due to the impossibility of post-trauma interventions from experts during more substantial impacts [54]. Nepal lacks resources, including human resource and services for mental health. Thus, the organizations involved were challenged to serve the colossal masses affected by the earthquakes. The strategies identified were mainly aimed at taking psychosocial support programs into communities as quickly as possible in a culturally and locally acceptable manner, which went forward under coordination with community-level workers.

Social cohesion, an essential aspect for community resilience [55], was identified by all organizations and was enhanced by keeping families together, conducting group tasks, and introducing community-led activities, which are also recognized strategies, as mentioned in one study [20]. Various age-appropriate local strategies [56] were adopted by organizations to make people feel normal in the distressing post-earthquake environment. Activities were carried out anywhere that was safe; whether within tents or within open grounds. “*We made a child friendly space*, *where children would come and play*, *draw*, *sing*, *dance*, *and learn about different things …. basically*, *it was aimed to give children a comfortable place and make them feel like home …. it also gave an opportunity for volunteers to observe children*”. —Organization 2. They mostly adopted locally drawn interventions to allow people to take control of their recovery process and restore their psychosocial capacity [57].

Although in Nepal there is a need to improve disaster preparedness and response efforts [46], interestingly enough, communities to some extent have built up their own resiliency through culture. Participants mentioned that the least affected groups shared helping hands with those severely affected by the earthquake in the post-earthquake phase. The Nepalese culture of taking care of each other, by offering help, strong communication, and problem sharing amongst families, friends, or neighbors, was mentioned as the strongest factor of resilience noted by participants within communities. They believe this culture played a more important role for lesser mental health impact than predicted. These cultural behaviors are thought to build trust amongst community members and to improve stress-coping capacity [7]. Communication and sharing helps communities with psychological recovery in the post-disaster phase [52].

### 3.3. Neglected Attention on Community Mental Health Resilience

Mental health is a low priority area and is stated to be present as only an additional part in policies at the service level. Organization 3 shared that, “*we can say it is mentioned only as a small part or in an integrated form everywhere*”. Despite slight increments after the earthquake, the low budget for mental health was a major highlighted issue that existed from the past. Organization 6 mentioned, “*let us say … if it was less than 1% before the earthquakes*, *now we can say it is more than 1% after the earthquakes … which is an improvement … however*, *we still have a long way forward*”.

Problems due to the absence of a proper structure for mental health services are commonly expressed by participants. Due to this absence, human resources from urban areas were gathered and mobilized to train local workers after the crisis. “*The current situation is …. existing community health workers are not even trained for mental health services … if they have the training*, *they could be immediately mobilized … like how there is a defined qualification and level of workers at the national level to sub-district levels for physical health care*, *there is nothing like that for mental health … in fact*, *those community workers are not even trained in any aspect of mental health*”.—Organization 4. Although some level of community mental-health capacity building was achieved after the earthquakes and previously unintroduced knowledge, skills, and awareness reached the community level through community-based programs, the coverage is still not enough for future preparedness.

Only non-governmental organizations in Nepal played significant roles in MHPSS training and intervention for many years, as support from the government for strengthening mental health in the country is not very strong [8]. A lack of a single focal body with a major spotlight on all aspects of psychological and mental health was expressed as an issue that led to a huge gap in service delivery. Organization 2 stated, “*mental health issues are monitored by the Health ministry*, *whereas psychosocial support is under the Department of women and children …. it is only in an integrated form … first*, *these two components should not be seen separately*; *and second*, *there should be a body that solely focuses on these issues*”.

There are no supporting guidelines or policies from the government for efforts carried out by various organizations in Nepal, as reported by participants. Similarly, a study mentions some improvements in the mental health policy but recognizes the limited implementation due to a lack of governance and leadership [10]. Various programs were carried out through the World Health Organization’s MHPSS component, which came forth under the humanitarian responses from the late 1980s [20]. However, they were found to be carried out on an ad-hoc basis, which is consistent with the findings from Sherchan et al. (2017) [15]. “*There is no such immediate activation guideline in Nepal … initially*, *organizations started doing whatever they felt was appropriate*”. —Organization 1. Some communities were reported to have received support within a short period, as compared to others, and conflicts arose due to the inequitable distribution. Preparedness was lacking and the initial response from the government was weak, but participants mentioned experiencing somewhat stronger coordination and monitoring of services in later stages. It was commonly mentioned that chaos in both relief and response delayed services for mental support to reach communities. Initially, services within communities were very random and uncoordinated. “*People with two days’ training also claimed they were counsellors or those with 10 days’ training also walked in as counsellors … everybody became a self-claimed counsellor*”. —Organization 8. However, only in the later stages were services checked for cultural appropriateness, the need for communities, quality, and duplication, after which the organizations stated they sensed a supportive environment to operate.

### 3.4. Social Stigma and Low Awareness on Mental Health

Various stigmas related to mental health preparedness problems were reported by participants. Mostly, people with mental disorders being looked down upon within society are a barrier felt by organizations for creating awareness as well as providing service. Organization 6 explained, “*They have a fear that people will call them mad (pagal)*”. Low awareness was not only a problem noted within communities, but also amongst different organizations that were active during the post-disaster phase. Organization 3 mentioned, “*many organizations would say … psychosocial supports are not necessary now …. those are for later*”. As communities became aware of benefits from services, they became more receptive to services and even sought out help. “*Previously mental illness issues did not come to the notice of health services*, *but now there is an increased number of visits and people seeking treatment*”. —Organization 2. This increased awareness in the aftermath of an earthquake is in line with the idea that disasters provide an opportunity for creating mental health awareness [13].

This study identified that communities lacked effective risk communication measures that would make them mentally prepared for the different situations arising during a disaster. Risk communication should be the focus before a disaster so that communities are aware of any impacts and how different actions may change the outcome of them. Information was identified as a major factor that was lacking within communities as well as among actors. Although some level of understanding was reported by actors, communities only had minimal information. Disasters are associated with emotional challenges in affected communities and the inability to understand their reactions or know about current disaster plans may indeed impact their mental health [58].

“*People did not know that hospitals would be inaccessible, roads could be blocked, there would be no electricity, phone network, water, and food … at first, people should be made aware that an earthquake does not just collapse buildings, but also significantly alters people’s lives. Therefore, they have to be prepared for an alternative lifestyle …. we should communicate with people on where to go, what to do, and whom to seek help from during that situation; this will show possibility and hope...if we can show a ray of hope, then resiliency starts building*”—Organization 5.

### 3.5. Mental Health Workforce and Service Accessibility Gap in Nepal

Even though mental health professionals play a strong role in tackling mental health impacts from earthquakes, the number of human resources in the country is significantly low; only 110 psychiatrists and 15 clinical psychologists are available for the entire nation [15], thus presenting a major barrier to better mental health outcomes, as mentioned by all organizations. For these 2015 earthquakes, the demand for the human resource was exceptionally high and service delivery was reported to be problematic, which prompted an immediate response to train competent people. “*When a situation like that arises*, *there should be human resources who are ready to be mobilized*; *for that*, *more mental health workers should be produced and retained in the country*”. —Organization 5.

Interview participants shared difficulties in transferring theoretical knowledge to practical situations, as staff members were unprepared and policies and plans did not work accordingly; these were similar to the issues identified in another study [9]. “*Although we had human resources*, *everyone was is a state of confusion on what to do and what not to do … we had guidelines*, *but the situation made everyone felt like*, *now what*”? —Organization 3. Even though disaster management was a highly discussed matter by most organizations, the actors themselves were not prepared for such a major catastrophe, which, in the future, demands more holistic and tangible disaster policies and plans. Many areas in Nepal are rural and unreachable due to a lack of transportation services. The challenge faced in providing services to affected areas, which are comparatively more developed than the western region of Nepal, led to alarming thoughts on what it would be like if disaster hits those remote areas. “*Providing services for cases of diarrhea and cholera to communities has itself been challenging to us … just imagine what would happen if we had to offer mental health services in those areas*”—Organization 5.

Even in scenarios where services were ready to be delivered, communities in rural areas were inaccessible. Organization 1 said, “*At that time due to landslides*, *rocks falling*, *and heavy rainfall*, *it was very difficult to reach affected communities*”. Circumstances in Nepal become worse during the rainy season and winter, making disaster-prone areas mostly inaccessible [9,59].

## 4. Discussion: The Way Forward

### 4.1. A Systematic Understanding of a Community’s Mental Health Needs

“*We understood their need, and through our community links, we have helped people to meet their basic needs … because it was important to make people get their basic needs fulfilled first …. that alone solved many problems I think*”—Organization 7.

People’s psychological needs may be an outcome from unmet physical needs during any crisis [60]. Hence, understanding the needs of an affected group is vital to produce better mental health outcomes. The promotion of various social support and resources [61] and ensuring a sense of stronger social support amidst a stressful atmosphere can help lessen the stress amongst affected communities [33], spur adaptation [33,35,39], and enhance resilience [33,35,39,62].

Our study’s significant concern is about the needs of vulnerable populations such as children, the elderly, and those severely affected low socio-economic groups who experienced the worst consequences and who live under extreme deprivation. One study listed women, unemployed people, rural dwellers, and people with low-self efficacy or mood and those with limited social support as making up the low resilience group [63]. Considering this, the government and different organizations should take active initiatives to identify the vulnerable populations and create a supportive environment during disasters to prevent serious consequences. The hierarchy of need for disaster survivors covers the following: (1) Food, water, and shelter, (2) safety, (3) family and friends’ support, (4) stress reactions, (5) grief and loss, and (6) assimilation/accommodation [64]. The needs depend upon the age and also the level of initial deprivation and inability to secure resources afterwards. Groups with higher losses may require a different level of assistance, whereas small children may require normalcy through basic needs as well as a secure environment and an opportunity to play, draw, sing, and engage in some learning.

Adults, on the other hand, may require support for regaining financial capabilities in later stages. While the loss of an income source might contribute towards psychological illness, economic aid programs such as job opportunities reduce stress [52] and enhance resilience in people [65]. Income and economic development may be a primal need for many affected communities in Nepal due to the immense scale of damage from the earthquakes; however, an emphasis on job opportunities was not pointed out by most interview participants and was not a priority of organizations at the initial stages. “*We focused on income generation after one year and realized it was more effective*”—Organization 6.

Introducing programs to encourage communities to generate income may be carried out by mental health workers in collaboration with financial and development workers, as suggested in a study [66]. However, the elderly population may benefit more from direct cash during disasters as compared to their younger counterparts, who require a more sustainable income source. Job security and assets are all crucial elements for adaptation after disaster impacts, for which securing financial sources before and after disasters is essential for disaster mental resilience. People with a low economic status are more likely to experience severe psychological consequences [33], due to difficulty in managing their household, handling stress over income and the change of lifestyle, and feeling worthless [66].

### 4.2. Improving Preparedness Capacity with Community-Based Programs

Disaster risk reduction should be a priority in Nepal by making Nepalese communities self-sufficient, because relying strongly on external resources to reach communities during a crisis makes them more vulnerable. There is a need to increase the mental health work-force to meet the service demand nation-wide. In the 2015 earthquake, communities could play an effective role in responding to disaster; however the scope and scale was limited, which indicates the need necessary to build capacity within communities [67]. Professional training for mental health and psychosocial support is identified as essential to contain the mental health impact by training communities and enhancing their capacity [68], from which they are also capable of recognizing vulnerabilities and strengths within them, which are extremely important for a community to be resilient [69].

An integrated community-based risk reduction (ICBRR) approach was implemented in Indonesia, which has enabled those communities to become the first responders to disaster risks [70]. Such community-based programs are incredibly relevant to rural and remote Nepalese communities, because they are mostly inaccessible due to geographical, infrastructural, or climatic challenges and human resource constraints, which are also the common excuses reiterated by the Nepal government for falling short in any disaster response [46]. Although coverage may be limited, various community-led activities were introduced during the earthquakes and are still carried on by volunteers, who bring communities together to identify issues and solve problems. “*FCHVs and mothers’ groups meet once a month and learn about different things*; *they can easily identify signs and symptoms and find ways to cope with problems*”—Organization 4. Such community-led services, in a way, make communities stronger, even though organizations are outside the realm; communities are now able to deal with similar issues to some extent. A similar kind of capacity building is needed in all areas that lack experience, especially in remote far western parts of Nepal, where thoughts of earthquakes around the same magnitude are alarming.

The government should understand the opportunities and capacities that they can utilize to prepare communities to face other disasters in the future. Although local community people are untrained, uninformed, inexperienced, and under-resourced to deal with disasters in Nepal [71], a certain level of resiliency building might have been possible during these earthquakes in areas where various services and activities were drawn together. A community-based approach targeting the capacities of communities and enabling them to run psychosocial support and skill-building programs for a large number of survivors [69] could be done by incorporating community-level resources such as schools and integrating community self-help groups and other local resources [72]. Teachers form a beneficial group of human resources in rural and remote communities. Teacher-based resilience-focused interventions, such as psycho-education, could be implemented in Nepal. This is a very cost-effective and universally available approach to prevent the development of problems like PTSD in children and families and to foster preparedness in school children facing trauma [54]. Furthermore, community capacity can be strengthened by facilitating effective risk communication in the face of a disaster, from which people are aware of what to do and where to go for assistance, making an entire community resilient during a disaster [73].

### 4.3. Promoting Resilience Through Religious and Spiritual Approach

O’Sullivan et al. (2013) argued that the concept “one size fits all” is inadequate for promoting resilience, as interventions should be suited to contextual factors [74]. By understanding the secure attachment of Nepalese towards religion and faith, this strategy may become a straightforward yet most powerful way to tackle long-term impacts and enhance their resilience. Various studies highlight the involvement of religious or spiritual activities [18,75,76] as essential for community mental resilience. This could also be effective in dealing with stigma issues that comes along with other mental health related services. Although some spiritual and religious involvements were informed by participants, strategies including these aspects were not prioritized enough following these earthquakes.

A recent study in Nepal investigated the relationship between religion and resilience and identified that participants who did not engage in any forms of prayer in the aftermath of an earthquake exhibited high scores for anxiety, depression, withdrawal, behavioural problems, and physical issues [18]. Another study conducted on the 2004 Asian tsunami in Tamil Nadu, India identified that spiritual coping (spiritual help-seeking, rituals) was the most commonly expressed factor for resilience amongst survivors [77]. Tuck and Anderson (2014) identified various studies where spirituality and resilience have a connection that favors mental health [3]. Communities are also relieved from stress to some extent through religious practices. “*After religious engagement*, *community members used to mention … religious chanting gave them peace*”*—*Organization 1. This method of coping is also in line with a study conducted on survivors of Hurricane Katrina in the U.S., where many expressed spiritual beliefs, in which their firm holding of religious ideas and faith in God was an integral aspect for coping with the disaster impact [75]. Religion may also be an effective strategy to discourage unhealthy behaviour, such as alcohol and drug use [76], as a some involvement in harmful coping strategies was mentioned during the interviews. “*Due to stress and sufferings*, *men started drinking alcohol and women started using tobacco*”. *—*Organization 1

Understanding the religious and spiritual aspects of Nepalese communities and building a partnership with faith-based organizations to optimize the capacity through existing resources could be effective paths going forward. Faith-based organizations played an influential role in providing relief and assistance during the Indian Ocean tsunami in 2004 [78]. They can play a significant role in psycho-social recovery by helping to reduce anxiety, providing comfort, and offering access to necessary resources and information [78]. Interventions from faith-based organization, support and religious book study groups, and community religious programs are effective solutions for a low-resource setting that depend highly on international assistance [79]. Therefore, the government and other organizations should consider incorporating religious and faith-based organizations as a significant partner for disaster response. The UNDP guidelines on engaging with faith-based organizations and religious leaders (2014) may be considered before building up such partnerships.

### 4.4. Improving Leadership and Governance of Mental Health Related Activities

Strengthening disaster response and preparedness is identified as the main improvement to be made in Nepal for better community mental resilience. The chaos, delay, insufficiency, and confusion identified in this study suggest a need for better response capacities. The present context, as understood from the disaster response to the 2015 earthquakes, revealed the low possibility of the country to deal with a future disaster without international assistance [46]. The massive impact from the 2015 earthquakes, followed by the slow-paced recovery actions and delayed adaptation to a post-earthquake environment, has gradually shown the negative influence on the mental health of the affected population as they become more disadvantaged over time [46]. Resources for psychosocial aspects, such as food, water, first aid, or shelter, are needed for sound social or community support systems to be set in place [73,80]. Improving access to services for good quality health contributes largely to socio-economic well-being and physical as well as psychological health [73]. There should be a facilitation of mental health-related resources to influence better mental health outcomes.

Various organizations are active during a humanitarian crisis and work according to their particular purpose. However, to draw maximum resources together for psycho-social restoration, multiple stakeholders should be able to function collaboratively with each other and scale up their common efforts. It is noteworthy that, during the earthquakes, it was more effective when communities were approached with more holistic services versus only mental health-specific services, thus signifying a need for establishing partnership amongst different organizations. “*People were not ready for mental support services until they got basic services*; *they would only run towards places where they would see the stockpiles of basic items*”. —Organization 3.

Different stakeholders from multiple backgrounds should work in partnership with each other for more effective response operations. A collaborative approach following an earthquake is extremely useful; therefore, the government should make plans and policies that include those different stakeholders. Chandra et al. (2013) suggested that a partnership between the government and non-government organizations and a careful integration of services bring along greater efficiency during disaster recovery [73].

### 4.5. Effective Risk Communication Through Relevant Information, Before, During, and After Disaster

Communities with adequate information are more resilient than those without information [33]. Having good sources of trusted, appropriate, and timely access to information [81] and offering a communication medium that instills hope and media support are argued as essential assets for community resilience [33]. It was mentioned by interview participants that false and misleading information brought chaos to communities, which added to the increased and prolonged stress in addition to that caused by frequent aftershocks. Providing support from correct and timely information reduces stress and uncertainty in communities [73]. During disasters, proper communication of messages through leaders and positive media influence is vital for building trust and positivity in communities [56]. Information reaching communities should be reassuring and reliable [56]. Thus, proper monitoring of the media and social networking site coverage is vital in the pre- and post-disaster phases.

Awareness through non-formal psychoeducation and disaster education can also be carried out for both adults and children. Since large numbers of children are severely affected [82] during a disaster, effective risk communication before a disaster is thought to lessen the mental health impacts [58]. Information may be fostered before a disaster through psychoeducation in school or through parents and other channels like advertising campaigns and social media, from which they can have a sense of the emotional changes and be aware about what to expect during a disaster [58]. Risk communication to underprivileged communities should be planned accordingly by using the correct medium for communication. Online media, print media, and radio and television media are present in Nepal and could play essential roles in ensuring that information reaches communities and that people are correctly informed and positively helped.

## 5. Conclusions

The current situation in Nepal reflects the need for mental health mainstreaming in all aspects of health and developmental tasks. Despite the colossal loss, this study found that the 2015 earthquakes shed light into the eyes of the Nepal government regarding the importance of mental health assistance during and following a disaster. By understanding the mental impact in the aftermath of the earthquake, there should be an independent focal body to deal with all aspects of psychological and mental health, instead of a mental health division that is just a small part of another governing body.

This study suggests that responses should be strengthened beforehand through policies and emergency preparedness drills and must not only be reactive. The path to better mental resilience may be challenging in the present context, but it is possible to achieve with suggested improvements and the proper utilization of existing resources. Strong societal and community bonds, which were identified as essential resilience factors in this study, could form the take-home message for other disaster-prone countries. The findings of this study can be implemented in other disaster-prone areas with a similar context.

While this research involved the perspective of acting organizations, future studies could focus on community members’ perspectives to suggest the necessary improvements, thus offering a more precise understanding on the topic by adding on to our findings. Future research could also evaluate to what extent strengthening adaptive capacities is possible through the efforts made during an earthquake. Currently, the need for recovery and helping communities get back to their regular days should be the basis for all implemented strategies to work out and requires tremendous commitment from the government. Such a strong commitment is also required to create an environment that enhances community mental resilience for future disasters through plans and policies that can be implemented before any disaster. Although barriers may be plentiful, there are still capacities within communities that should be recognized and utilized for better mental health outcomes.
